# Supplementary material for: S100A8 and S100A12 Proteins as Biomarkers of High Disease Activity in Patients with Rheumatoid Arthritis That Can Be Regulated by Epigenetic Drugs
Source: Int J Mol Sci. 2022 Dec 31;24(1):710. doi: 10.3390/ijms24010710 (PMC9820830; doi:10.3390/ijms24010710)
Supplement: Supplementary file 1 [file ijms-24-00710-s001.zip › ijms-2052018-supplementary.pdf]

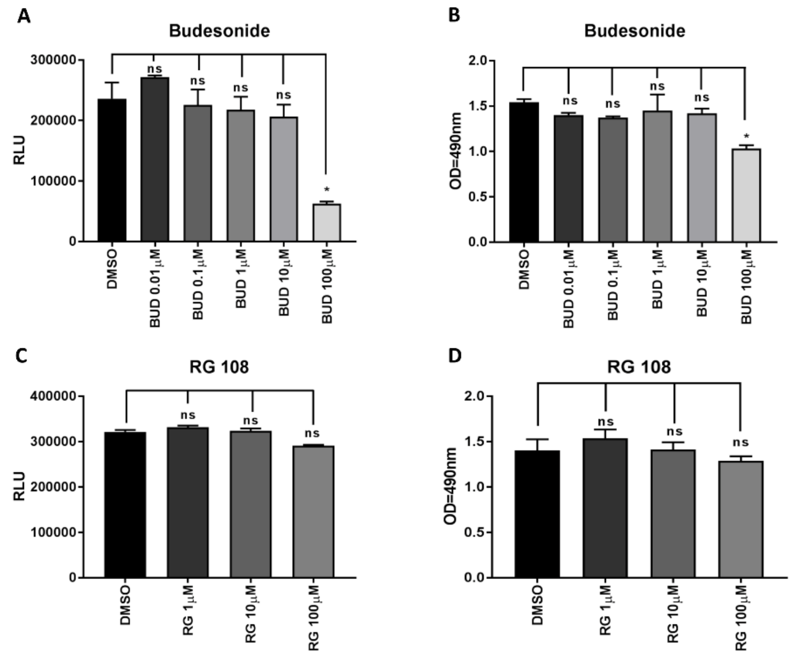

Figure S1. Viability of the THP-1 monocytic cell line measured by the chemiluminescence (**A,C**) and by the MTS assay (**B,D**) after treatment with graded concentrations of budesonide (**A,B**) and RG 108 (**C,D**) following 24-hour incubation (n = 3–6). P values were expressed as follows: 0.05 > p > 0.01 as * and ns – not significant.

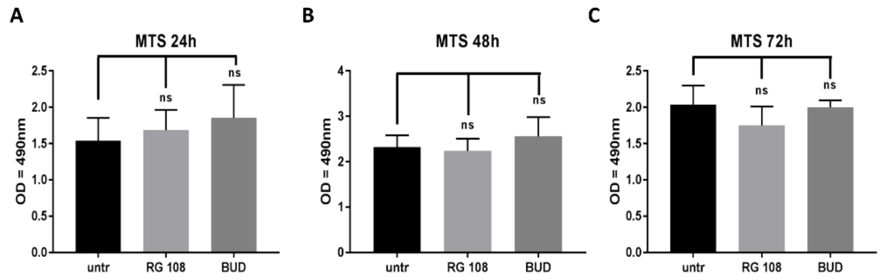

Figure S2. Viability of the THP-1 monocytic cell line measured by the MTS assay after treatment budesonide (10 µM) and RG 108 (100 µM) after different time points 24 (**A**), 48 (**B**) and 72 hours (**C**) (n = 5). ns – not significant.

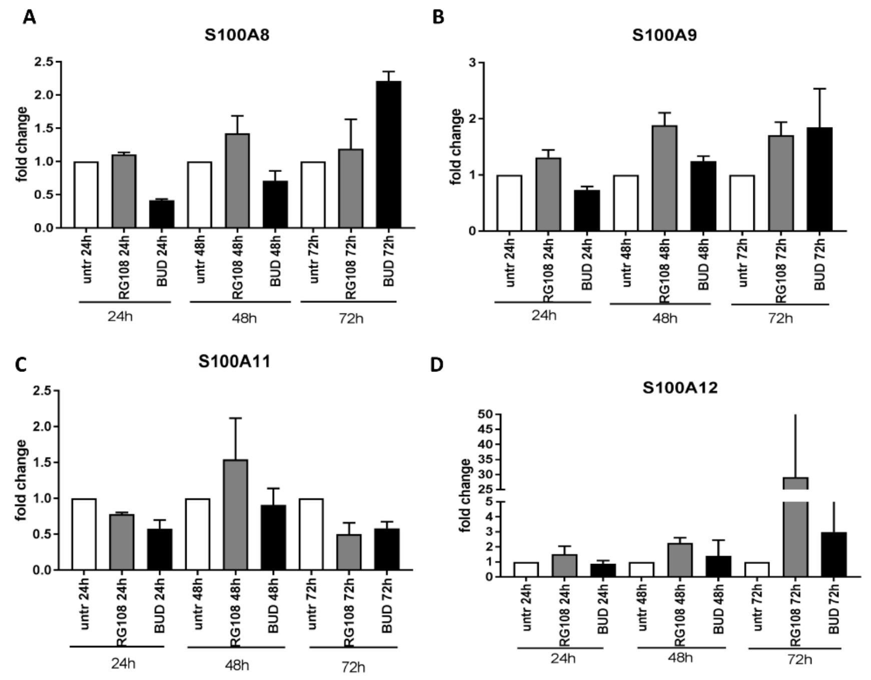

Figure S3. Expression level of S100A8(**A**), A100A9(**B**), S100A11(**C**) and S100A12(**D**) followed by budesonide or RG108 treatment in THP-1 cells measured by qRT-PCR at different time points (n = 2).
